# Changes in ppGpp levels impact gene expression and virulence features of Adherent-Invasive *Escherichia coli* strain LF82

**DOI:** 10.1016/j.crmicr.2026.100641

**Published:** 2026-07-07

**Authors:** Llorenç Fernández-Coll, Queralt Bonet-Rossinyol, Mireia López-Siles, Katarzyna Potrykus, Maria Núria Ramos-Corominas, Margarita Martinez-Medina

**Affiliations:** a Microbiology of Intestinal Diseases, Biology Department, University of Girona, Girona, Spain; b Department of Genetics, Microbiology and Statistics, University of Barcelona, Barcelona, Spain; c Department of Bacterial Molecular Genetics, Faculty of Biology, University of Gdansk, Gdansk, Poland

**Keywords:** Adherent-Invasive *Escherichia coli* (AIEC), ppGpp, Transcriptomics, Virulence, *Galleria mellonella*, Biomarkers

## Abstract

Strain LF82 belongs to the Adherent-Invasive *Escherichia coli* (AIEC) pathotype, found in 21% to 63% of Crohn’s disease (CD) patients. Many studies have explored its specific virulence mechanisms, but AIEC isolates are genomically similar to non-invasive isolates of *E. coli*, suggesting a differential gene expression. Here we study the impact of the global regulator ppGpp in the expression of AIEC’s virulence factors and its potential for therapeutic and diagnostic purposes. Levels of ppGpp were determined from P-33 labelled cultures of LF82 and MG1655 (commensal strain) and differences in gene expression during infection of intestinal epithelial cells were assessed through RNAseq. Our study revealed that ppGpp is responsible for the expression of different genes involved in AIEC’s pathogenicity (Type I fimbriae, T6SS or Vat), and other global regulators (as CRP or c-di-GMP). AIEC characteristics and virulence features (biofilm formation and motility) were determined, showing that ppGpp is essential for LF82 to adhere to and invade epithelial intestinal cells, as well as to survive in macrophages. Lastly, virulence was evaluated *in vivo* using the *Galleria mellonella* larvae model, showing a decrease in virulence in the absence of ppGpp. Analysis of a panel of differentially expressed genes across a collection of 7 AIEC strains and 6 non-AIEC strains, identified two candidate biomarkers which expression show a discriminatory potential for AIEC identification in the future. Altogether, our findings establish ppGpp as a key regulator of the AIEC virulence program and provide novel targets for both therapeutic intervention and diagnostic development.

## Highlights

• The global regulator ppGpp plays a central role in controlling AIEC virulence.
• Loss of ppGpp impairs virulence across *in vitro* and *in vivo* infection models.
• Two candidate biomarkers with discriminatory potential for AIEC identification were defined.

## Graphical abstract

**Figure:**
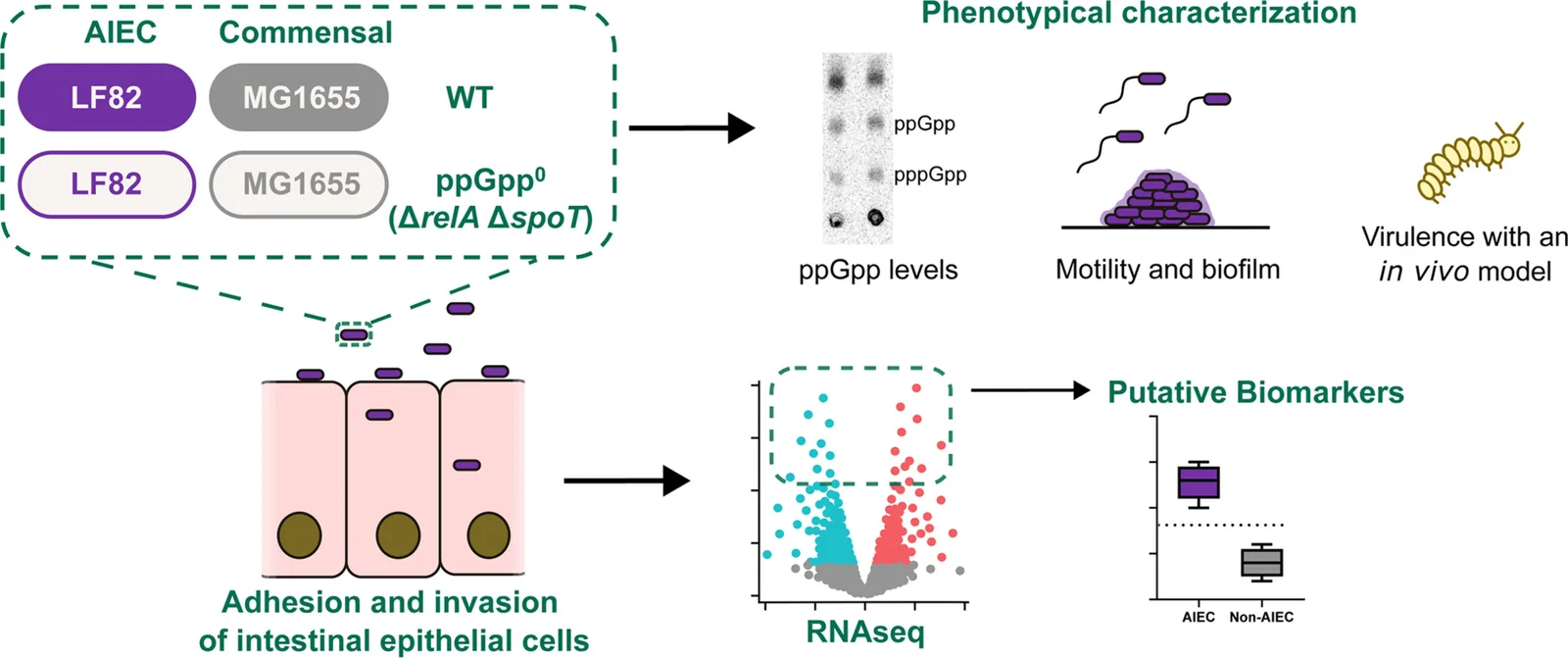

## 1 Introduction

Adherent-Invasive *E. coli* (AIEC) is a pathobiont associated with Crohn’s Disease (CD) (Baumgart et al., 2007; Darfeuille-Michaud et al., 2004; Martinez-Medina et al., 2009), a chronic-relapsing inflammatory bowel disease (IBD) that can involve the entire gastrointestinal tract. CD affects up to 0.2% of the European population and incidence grew in the last years (Zhao et al., 2021). The etiology of CD is unclear, but genetic and environmental factors, together with microbiota dysbiosis, have been associated with this disease (Mirsepasi-Lauridsen et al., 2019). AIEC infection can explain several features of the pathophysiology of this disease, such as inflammation, mucosal translocation, and the formation of granulomas (Palmela et al., 2018). These strains are characterized by their ability to bind to and invade intestinal epithelial cells (Boudeau et al., 1999), as well as to replicate inside macrophages (Glasser et al., 2001). No common virulence factors were described in AIEC, but they lack those virulence factors that define other *E. coli* intestinal pathotypes (*i.e.*, EIEC). Currently, there are no biomarkers to identify this pathotype.

The discovery of clonally identical *E. coli* isolates with identical pulsotypes but differing in the AIEC phenotype (Martinez-Medina et al., 2009), suggested that differential gene expression, rather than genomic content, underlies AIEC traits (Camprubí-Font et al., 2018). Transcriptomic analysis of AIEC / non-AIEC strain pairs during infection of intestinal epithelial cells revealed differential expression of genes involved in amino acid biosynthesis and fimbriae (Bonet-Rossinyol et al., 2023). Several of these genes are regulated by the global regulator (p)ppGpp (Aberg et al., 2008; Wang et al., 2020), suggesting a possible dysregulation of ppGpp synthesis or its activity in AIEC.

The penta- and tetra-phosphate of guanosine, (p)ppGpp are global regulators of bacterial gene expression found throughout the bacterial kingdom and even in plant chloroplasts (Irving et al., 2021; Mehrez et al., 2023). Both are accumulated during stress (Cashel and Gallant, 1969), but pppGpp seems to be less potent than ppGpp in *E. coli* (Mechold et al., 2013) and it is usually transformed into ppGpp by the enzyme GppA. Thus, these effectors are often abbreviated simply as ppGpp. In *E. coli*, ppGpp is synthesized by the RelA protein during amino acid starvation and by the bifunctional enzyme SpoT in response to different stress and starvations (Potrykus et al., 2026; Potrykus and Cashel, 2008). SpoT is also the sole hydrolase of ppGpp found in *E. coli*, which can change conformation between hydrolysis and synthesis depending on the environmental conditions (Hogg et al., 2004). Once accumulated, ppGpp binds to the RNA polymerase (Mechold et al., 2013; Molodtsov et al., 2018; Ross et al., 2016) and alters gene expression to inhibit bacterial growth and activate stress-survival pathways (Magnusson et al., 2005). Its effects depend on concentration and accumulation speed (Fernández-Coll and Cashel, 2020) and are further modulated by transcription factors binding the RNA polymerase secondary channel, such as DksA or the Gre factors (Aberg et al., 2009; Fernández-Coll et al., 2018; Vinella et al., 2012).

In a study determining the important factors for AIEC’s ability to survive inside macrophages, ppGpp was found to be essential for its survival (Demarre et al., 2019). However, we have no knowledge of the role that this regulator may have during infection of intestinal epithelial cells or its effect on the gene expression patterns of AIEC strains. Thus, in this study we aim to determine the effect of ppGpp on the virulence of the AIEC strain LF82 using *in vitro* and *in vivo* infection models. Moreover, we aim to study the effect of ppGpp on the gene expression pattern of LF82 compared to the commensal strain MG1655 during the infection of human enterocytes. We believe that this study may unravel new factors involved in AIEC’s pathogenicity that could be used as biomarkers of this pathobiont.

## 2 Materials and methods

### 2.1 Strains and media

Strains used in this study are listed in Table S1. The ppGpp-deficient strains (*i.e.* LF82 ppGpp^0^ and MG1655 ppGpp^0^) were obtained by deleting *relA* and *spoT* through the λ-red recombination method (Datsenko and Wanner, 2000). Primers relA-P1/relA-P2 and spoT-P1/spoT-P2 were used to amplify the Cm and Km resistant genes from pKD3 and pKD4, respectively (Table S2). Bacteria were grown routinely in LB at 37 °C with shaking (200 rpm), but other media were used when indicated, such as minimal medium M9 (Delmas et al., 2019) or CFA (Paytubi et al., 2017).

### 2.2 (p)ppGpp measurements

(p)ppGpp levels were measured by P-33 labelling and separation by thin-layer chromatography (TLC) as previously described (Fernández-Coll and Cashel, 2019; Nishino et al., 1979). Amino acid starvation was induced with 1.5 mg/ml of serine‑hydroxamate (SHX), a structural analogue of L‑serine. To measure the % of (p)ppGpp, the radioactive spots were quantified with ImageJ (Schindelin et al., 2012; Schneider et al., 2012) and the amounts of ppGpp and pppGpp were normalized to the total amount of radioactive signal (pppGpp + ppGpp + GTP) for each sample. The experiment was done in triplicate (biological replicates).

### 2.3 Biofilm formation

Formation of biofilm was assessed as published before (Tajuelo et al., 2023) with slight modifications. Briefly, overnight (ON) cultures were diluted to 10^8^ CFU/ml in CFA in 96-well plates (U-shaped) and incubated statically 72h at 30 °C. Then the biofilm was stained with crystal violet, washed with PBS to remove excess dye and measured at OD596nm. Data was normalized to OD600nm before staining to calculate the specific biofilm formation index. Experiments were performed with 3 biological and 3 technical replicates.

Production of curli fimbriae and cellulose was assessed based on RDAR (red, dry and rough) phenotype in Congo Red (CR) plates (Simm et al., 2014). Stationary phase cultures (OD600nm ≈ 2.0) of LF82 and LF82 ppGpp^0^ were spotted (10 µl) at the surface of the CR plate and incubated at 37 °C for 24h.

Mannose-sensitive yeast agglutination assay (MSYA) was performed as previously published (Aberg et al., 2006). Presence of aggregates was qualitatively scored.

### 2.4 Motility assay

Swimming motility was assessed as previously described (Aberg et al., 2009). No chemo-attractants or repellents were added to the motility agar. Plates were incubated for 8h at 37 °C, then the diameter of the colony was determined, and the area was calculated. For each strain, 3 biological replicates with 4 technical replicates were performed.

### 2.5 Adhesion and invasion assay

Bacterial adhesion and invasion of epithelial cells was determined using Intestine-407 epithelial cell line (I-407; ATCC CCL-6) as previously described (Boudeau et al., 1999). I-407 cells were grown in Minimum Essential Medium (MEM) supplemented with 10% inactivated FBS, 2 mM L-Glutamine, non-essential amino acids, vitamin solution and antibiotic-antimycotic solution (Gibco). Briefly, duplicated plates of I-407 monolayers (4·10^5^ cells/well) were infected with a MOI of 10 and incubated for 3h at 37 °C with 5% CO_2_. To measure cellular adhesion, cell monolayers were washed 3 times with DPBS, lysed with 1% Triton X-100, and plated on LB agar plates. The adherence index (ADH_I) was calculated as the mean number of bacteria per cell. To measure cellular invasion, cell monolayers were washed 3 times with DPBS, fresh MEM media containing 100 µg/ml of gentamycin were added and incubated for an hour at 37 °C to kill any extracellular bacteria. After that, cell monolayers were washed again (3 times), were lysed with 1% Triton X-100, and plated in LB agar. Invasive index (Inv_I) was determined as the percentage of intracellular bacteria compared to the inoculated bacteria.

### 2.6 Intramacrophage survival assay

Survival ability and replication inside murine macrophage cell line J774A.1 (ATCC TIB-67) was determined as previously described (Glasser et al., 2001). J774 monolayers containing 4·10^5^ cells/well were infected with a MOI of 100, centrifuged (10 min at 900 RPM) to promote contact between macrophages and bacteria and then incubated for 10 min at 37 °C with 5% CO_2_. After that, gentamicin 20 µg/ml was added for 40 min (T_1_) or 24h (T_24_) to eliminate bacteria present outside macrophages. Macrophages were lysed with 1% Triton X-100, diluted and plated in LB agar. The strains were considered to survive when the percentage of intracellular bacteria at T_24_ relative to intracellular bacteria at T_1_ was ≥100%, and to replicate within macrophages when it was ≥200%.

### 2.7 *In vivo* infection of *galleria mellonella*

ON cultures (25 ml) of LF82 and LF82 ppGpp^0^ were washed 3 times with saline solution (0.9% NaCl (w/v)) and adjusted to a concentration of, 10^10^ CFU/ml. Then, the cultures were serially diluted saline solution to obtain suspensions from ∼5·10^9^ to ∼10^7^ CFU/ml and plated to determine the number of CFU inoculated.

Wax moth larvae (*Galleria mellonella*) were purchased from local providers and were maintained on wood chips in the dark at room temperature. Groups of 10 *G. mellonella* larvae in the sixth instar stage were inoculated with 10 µl of each bacterial solution in the second rear proleg using a Hamilton syringe. A set of larvae was also injected with 10 µl of saline solution as a control group. After 24h, the number of dead larvae (lack of movement and dark pigmentation) was determined and the percentage of lethality calculated. Data represented was gathered in 3 independent experiments.

### 2.8 Survival under oxidative stress

Strains LF82, LF82 ppGpp^0^, MG1655 and MG1655 ppGpp^0^ were grown to OD600 nm of 0.5 and then H_2_O_2_ 2 mM was added to the cultures. Samples were taken at 0, 20 and 40 min after adding H_2_O_2_ and the number of CFU was determined. The survival rate was expressed as the percentage of the number of bacteria after 20 and 40 min compared to the number of bacteria from the initial time point. This study was performed with 3 biological replicates.

### 2.9 Survival when exposed to acid and nutrient-poor conditions

ON cultures of LF82, LF82 ppGpp^0^, MG1655 and MG1655 ppGpp^0^ were adjusted to an OD600nm of 0.1 in acid and nutrient-poor medium (Bringer et al., 2005) containing 100 mM Bis-Tris, 0.1% casamino Acids, 0.16% glycerol, and 10 μM MgCl_2_, adjusted to pH of 4.5 with HCl. The number of CFU was determined at time 0 and after 24h incubation at 37 °C. The survival rate was determined by comparing the number of bacteria after 24h to the CFU at time 0. This experiment was performed with 3 biological replicates.

### 2.10 Minimum inhibitory concentration (MIC) determination

ON cultures of LF82, LF82 ppGpp^0^, MG1655 and MG1655 ppGpp^0^ were adjusted to a concentration of 10^5^ CFU/ml and incubated at increasing concentrations of lysozyme and Polymyxin B (PmB) – from 50 to 0.1 µg/ml – and Polymyxin E (PmE) – from 10 to 0.02 µg/ml. The minimum inhibitory concentration (MIC) was determined after 24h at 37 °C. This experiment was performed with 3 biological replicates.

### 2.11 RNA isolation from infected intestinal epithelial cells

RNA isolation was performed as previously published (Bonet-Rossinyol et al., 2023). Briefly, T75 flasks of I-407 monolayers with 2·10^7^ cells were infected with bacterial cultures of each strain (by triplicate), at exponential phase with a MOI of 100. After 4h incubation at 37 °C with 5% CO_2_, the supernatant (SNT) and the cellular fractions were collected, pelleted by centrifugation, washed with DPBS and frozen at −80 °C until RNA was extracted. RNA was isolated using TRIzol (Invitrogen), followed by the degradation of any leftover genomic DNA with the TURBO DNA-free™ Kit (Invitrogen), following manufacturer recommendations in both cases. Eukaryotic RNA was depleted using MICROBEnrich kit (Invitrogen) and the prokaryotic rRNA was also depleted with the Illumina Ribo-Zero Plus rRNA Depletion kit.

### 2.12 RNA sequencing

RNA sequencing was performed as previously published (Bonet-Rossinyol et al., 2023). RNAseq and bioinformatic analyses were carried out by AllGenetics & Biology SL (www.allgenetics.eu). The quality of the RNA samples was determined with an Agilent, 2100 Bioanalyzer using the Agilent RNA, 6000 Nano Kit, before preparing the paired-end libraries with the NEBNext® Ultra™ II Directional RNA Library Prep Kit (New England BioLabs). Samples were sequenced on a NovaSeq PE150 flow cell (Illumina) aiming for a total yield of 380 gigabases. The quality of the FASTQ files was assessed with the FastQC software (Andrews, 2010), and Trimmomatic v0.39 (Bolger et al., 2014) was used to remove the adapters (ILLUMINACLIP option), low-quality regions (AVGQUAL:26) and remaining reads shorter than 80 bp (MINLEN:80). We used STAR 2.7.8a (Dobin et al., 2013) to align the retained reads to the *E. coli* LF82 assembly (GenBank accession number: GCF_021398935.1) or to the *E. coli* K-12 substr. MG1655 (GenBank accession number: GCF_000005845.2). Read counts were computed using HTSeq-counts v0.13.5 (Anders et al., 2015) and normalized and filtered with Trimmed Mean of M-values (TMM) method (Robinson and Oshlack, 2010). The differential expression analysis was conducted using the Bioconductor packages NOISeq and NOISeqBIO (Tarazona et al., 2015), with a probability threshold of q = 0.90. The variability between replicates in each sample was determined with a Principal Components Analysis (PCA) during comparisons of the SNT and cellular fraction (Fig S7). The differentially expressed genes were classified according to the COG’s classification (Galperin et al., 2021; Tatusov et al., 2000).

### 2.13 RT-qPCR

The expression of *iraP* was determined by RT-qPCR. Briefly, ribo-depleted RNA was retrotranscribed into cDNA using the High-capacity cDNA Reverse Transcription kit (Applied Biosystems) and the target gene was amplified with SYBR Green PCR Master Mix (Applied Biosystems) using primers iraP-FW / iraP-RV (Table S2). The relative gene expression was determined with the comparative CT method, also known as 2^–ΔΔCt^ (Schmittgen and Livak, 2008) using the gene *adk* as housekeeping control, amplified using the primers adk-FW / adk-RV (Table S2).

### 2.14 Fluidigm

RNA isolated previously (Bonet-Rossinyol et al., 2023) from 7 AIEC (AIEC04, AIEC07, AIEC11, AIEC14, AIEC17, AIEC23 and AIEC25) and 6 non-AIEC (ECG01, ECG04, ECG15, ECG17, ECG18 and ECG28) strains during infection of I-407 monolayers (SNT and cellular fraction) was retrotranscribed using the High-capacity cDNA Reverse Transcription kit (Applied Biosystems). These strains were previously isolated from ileum and colon biopsies from IBD patients and control subjects (Martinez-Medina et al., 2009). The RT-qPCR was performed using an IFC 96 × 96 Evagreen microfluidic array chip on a BioMark™ system (Fluidigm, USA). Data analysis was performed as before (Bonet-Rossinyol et al., 2023), where the relative transcript abundance (RTA) was normalized to the expression levels at LF82. Three technical replicates were analyzed for each sample. The list of primers used to amplify the selected genes can be found in Table S2. As before, the gene *adk* was used as housekeeping control.

### 2.15 Data analysis and statistics

**Fig. 1:**
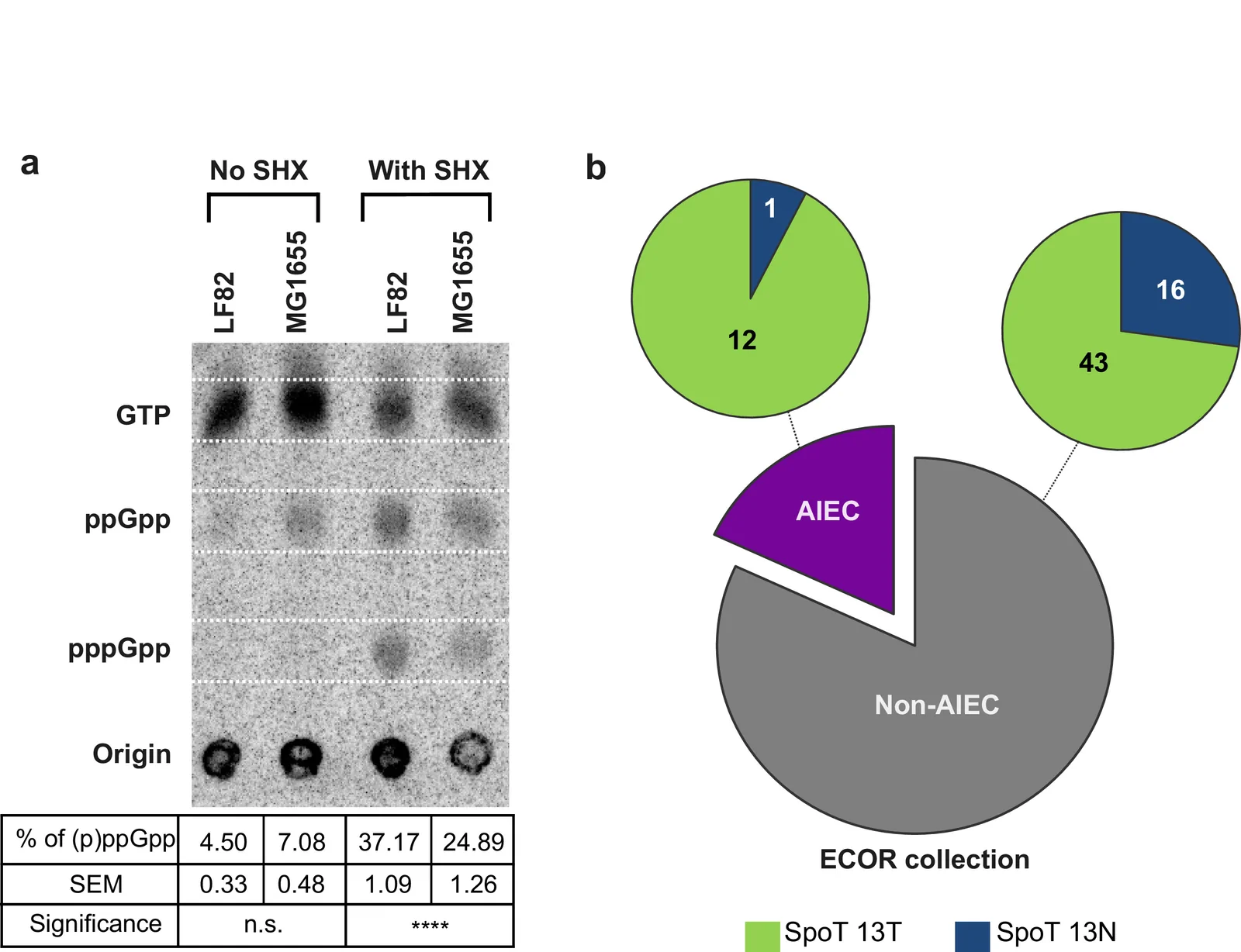
**Levels of ppGpp in LF82 and MG1655.** a) The levels of ppGpp and pppGpp were determined from P-33 labelled cultures of LF82 and MG1655 before and after adding serine‑hydroxamate (SHX). A representative thin-layer chromatography (TLC) is shown. Mean and standard error (SEM) of % (p)ppGpp (spot quantification of ppGpp and pppGpp relative to the total amount of signal detected) is indicated below. This experiment was performed in triplicate (3 biological replicates). A T-test was used to determine those values significantly different (**** p-value 〈 0.0001, n.s. p-value 〉 0.05). b) Distribution of the SpoT alleles in the ECOR collection, separating those AIEC to non-AIEC strains.

**Fig. 2:**
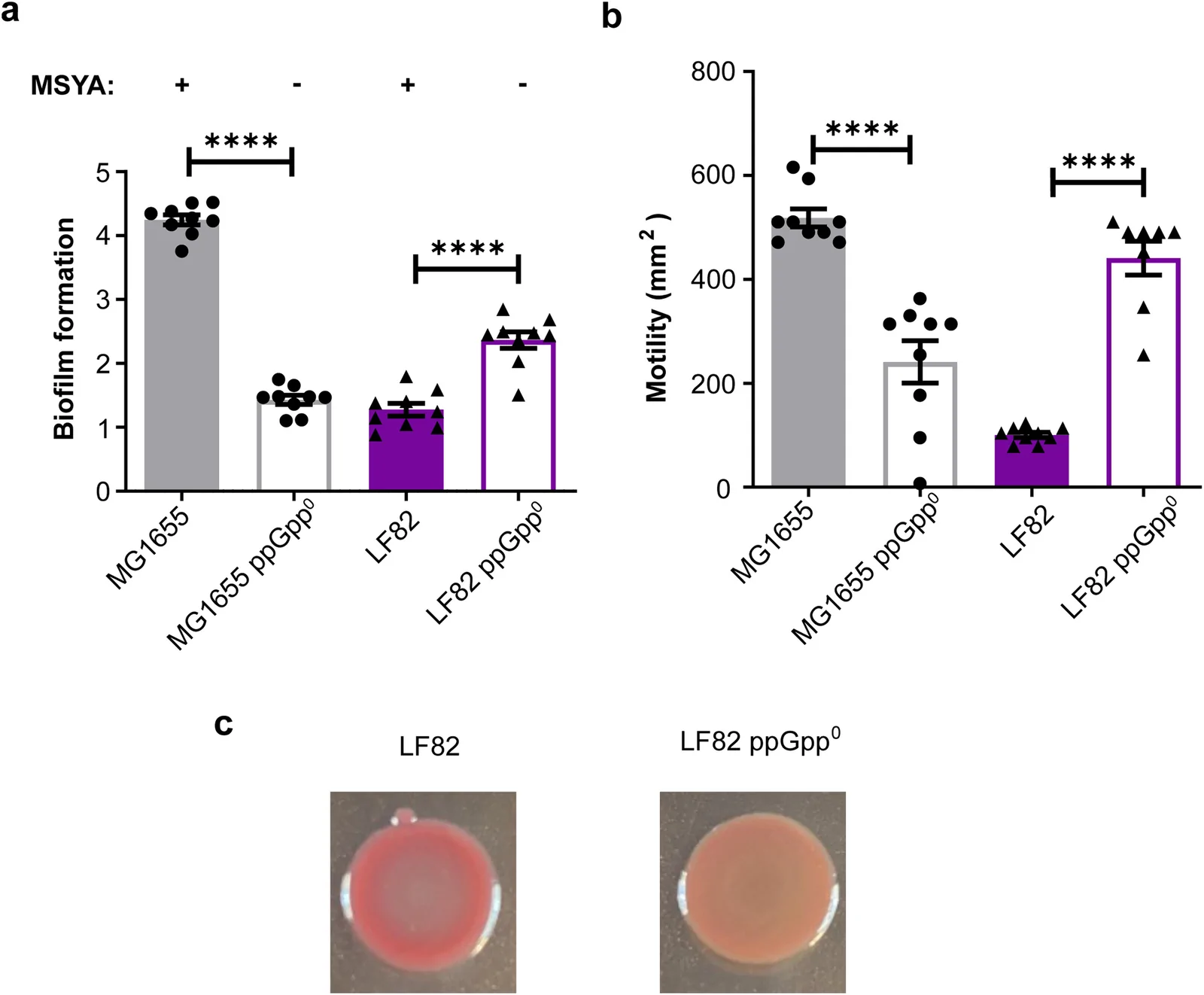
**Phenotypical characterization of the ppGpp-deficient strains.** a) Biofilm and type-I fimbriae formation and b) motility of MG1655, LF82 and their ppGpp^0^ mutants were assessed. Mean (with data points) and standard error (SEM) of 3 biological replicates is represented. Mannose-sensitive yeast agglutination (MSYA) assay was used to assess formation of type-I fimbriae and the presence of agglutination was scored – when absent and + when present. To determine those values significantly different, a T-test was performed in panel a, while Mann-Whitney was performed in panel b (**** p-value < 0.0001). c) Images of the macrocolonies produced by LF82 and LF82 ppGpp^0^ in CR (Congo Red) plates are shown.

**Fig. 3:**
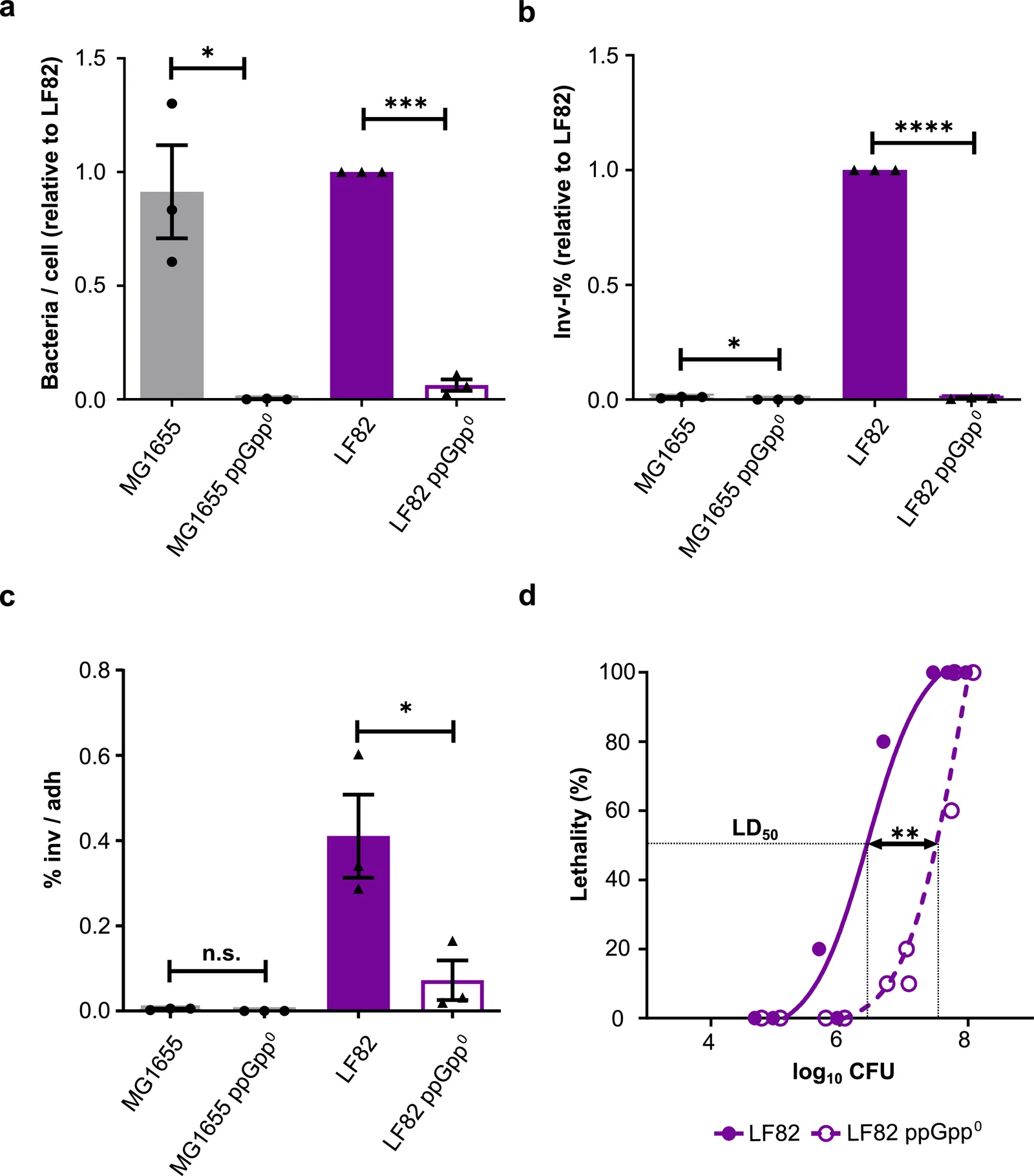
**Effect of ppGpp on AIEC virulence.** Ability of the strains MG1655, MG1655 ppGpp^0^, LF82 and LF82 ppGpp^0^ to a) adhere to and b) invade to epithelial intestinal cells I-407. c) Percentage of bacteria invading I-407 normalized to those bacteria able to adhere to epithelial cells. Mean (with data points) and standard error (SEM) of 3 biological replicates is represented. d) LD_50_ of LF82 and LF82 ppGpp0 strains in the *in vivo* model of *Galleria mellonella*, measured from a nonlinear regression with a goodness of fit (R2) of 0.956 and 0.942 for LF82 and LF82 ppGpp^0^, respectively. A T-test was used to determine those values significantly different, but Welch correction was applied to panels a to c (**** p-value < 0.0001, ** p-value < 0.01, * p-value <0.05, n.s. p-value > 0.05).

Normality (Shapiro-Wilk test) and homoscedasticity (Fisher’s F-test of equality of variances or Bartlett’s test) were assessed by data presented in Fig. 1, Fig. 2, Fig. 3 and Fgures S1-S3. Data that showed a normal distribution and homoscedasticity, a two-tailed T-test or one-way ANOVA were used to test differences in variables with 2 or >2 categories. Data with a normal distribution but heteroscedasticity, a Welch correction was applied to the Student’s *t*-test. Data without a normal distribution, Mann-Whitney U test was used. Fisher’s exact test was performed to determine any correlation between AIEC phenotype and SpoT alleles (Fig. 1b). To determine LD_50_ (Fig. 3d), a nonlinear regression was performed, and a two-tailed T-test was used to determine differences between the calculated LD_50_. Kruskal-Wallis test with multiple comparisons and Mann-Whitney test were used to determine differences of gene expression between AIEC and non-AIEC strains at different fractions or using the ratio between fractions (Fig. 7, Table S12). These statistical analyses were performed using the GraphPad v8 (Prism). The binary logistic regressions (Table 2) were performed with R. Significance levels were established for p-values < 0.05.

## 3 Results

### 3.1 Levels of ppGpp vary between MG1655 and LF82

First, we compared the levels of (p)ppGpp (both pppGpp and ppGpp together) between the AIEC strain LF82 and the commensal strain MG1655 (Fig. 1a). Amino acid starvation, induced with serine hydroxamate (SHX), increases the levels of (p)ppGpp rapidly in both strains, as expected (Cashel and Gallant, 1969). However, the levels of (p)ppGpp differed between LF82 and MG1655: while no differences were observed under basal conditions (no SHX), the levels of (p)ppGpp are significantly higher in LF82 (increases up to 8-fold compared to basal levels) than in MG1655 under amino acid starvation (increases 3-fold compared to the basal level).

Due to the increased accumulation of (p)ppGpp in LF82 compared to MG1655 under amino acid starvation, the sequence of both ppGpp synthetases (RelA and SpoT) were compared between both strains. No differences were observed in RelA, but a mutation near the hydrolase domain of SpoT was found in LF82: SpoT T13N. This allele (SpoT 13 N) was previously described in the ECOR collection – 72 environmental isolates from *E. coli* (Ferenci et al., 2011) – that contain 13 strains classified as AIEC (Rahmouni et al., 2018). The allele SpoT 13 N was found in 17 strains, most of them classified as non-AIEC and one classified as AIEC (Fig. 1b). No correlation between the SpoT alleles and the AIEC classification was observed using Fisher’s exact test (p-value = 0.1703).

### 3.2 Phenotypical characterization of ppGpp-deficient mutant strains

After deleting *relA* and *spoT* from LF82 and MG1655, the growth rate of the ppGpp-deficient mutants (ppGpp^0^) together with their parental strains (WT) was determined under different conditions (Table S3). In either MG1655 or LF82, the ppGpp^0^ mutant strains show similar growth to the WT in LB at 37 °C. We also determined its growth in M9 minimal medium, since ppGpp-deficient strains cannot grow in minimal medium without amino acids (Xiao et al., 1991). As previously observed for MG1655, the LF82 ppGpp^0^ strain does not grow in M9 minimal medium without amino acids (Table S3).

Considering that ppGpp is required to produce biofilm and swimming motility of the strain MG1655 (Aberg et al., 2009, 2006), we checked the role of ppGpp for these phenotypes in LF82 (Figs. 2a and b). In the ppGpp-deficient LF82, an increase in the formation of biofilm (Fig. 2a) and motility (Fig. 2b) was observed, suggesting that ppGpp acts as a repressor of production of biofilm and motility in LF82. As previously observed, MG1655 ppGpp-deficient strains showed a significant 3-fold reduction in production of biofilm (Fig. 2a) and 2-times less motility (Fig. 2b), validating our results. Thus, it suggests a differential regulation by ppGpp between the commensal MG1655 strain and the AIEC strain LF82. The mannose-sensitive yeast aggregation (MSYA) assay was positive in both WT strains (MG1655 and LF82), but negative in the absence of ppGpp (Fig. 2a), indicating a lack of functional type I fimbriae. These results suggest that ppGpp promotes expression of type I fimbriae in LF82, consistent with previous observations in MG1655 (Aberg et al., 2006).

The ability of LF82 and its ppGpp^0^ mutant to produce curli and cellulose was also assessed observing the RDAR phenotype in Congo Red (CR) plates (Fig. 2c), where pigmentation of the macrocolonies reflects the presence of both factors (red colonies), just production of curli (brown colonies) or the absence of both (white colonies). LF82 colonies are red suggesting the presence of both factors, while the colonies of LF82 ppGpp^0^ are brown, suggesting that those are only producing curli (Fig. 2c). Thus, our data suggests that ppGpp is important for the synthesis of cellulose, but not that much on the production of curli.

### 3.3 Effect of ppGpp on the AIEC features

As previously indicated, AIEC strains are characterized by their ability to adhere to and invade intestinal epithelial cells, as well as to survive and replicate in macrophages (Boudeau et al., 1999; Glasser et al., 2001). Thus, the ability of the WT and the ppGpp^0^ strains to bind and invade I-407 epithelial cells was determined (Fig. 3), as well as their ability to survive and replicate inside J774 macrophages (Fig. S2a).

Both WT strains, MG1655 and LF82, can bind epithelial cells, while the adhesion of the ppGpp^0^ strains was dramatically affected (Fig. 3a). In the absence of ppGpp, a 14-fold and a 600-fold decrease in adhesion was observed for LF82 and MG1655, respectively. Despite that, in the ppGpp-deficient strains we still detect approximately 6·10^5^ and 10^4^ bacteria attached to the monolayer, respectively. As for their ability to invade (Fig. 3b), only LF82 is capable of invading epithelial cells, while no invasion was observed for MG1655. The LF82 ppGpp^0^ mutant showed a 150-fold decrease in invasion compared to the WT counterpart.

Plotting invasion indexes alone, it is difficult to assess if ppGpp specifically affects invasion or if the reduced number of intracellular bacteria is due to lower adhesion. Thus, we plotted the percentage of bacteria able to invade related to those that adhered to I-407 cells (% inv / adh, Fig. 3c). We clearly observed a decrease on the percentage of invading cells in LF82 ppGpp^0^, compared to LF82, indicating an impact of ppGpp on both invasion and adhesion. Of note, no differences in growth rate were observed between the strains grown in the conditions of this experiment (statically incubated in MEM at 37 °C with 5% CO_2_, S3 Table).

In the absence of *relA, E. coli* cannot synthesize ppGpp in response to amino acid starvation, but ppGpp can still be produced in response to other stress conditions (Lazzarini et al., 1971) through SpoT. Deletion of *relA* in LF82 resulted in a 2-fold decrease in adhesion (Fig. S1a) and a slight decrease (but significant) in the ability of LF82 to invade epithelial cells (Fig. S1b). However, when invasion was normalized to the number of adhered bacteria, the invasion percentage is slightly higher in the mutant than the WT strain (Fig. S1c), suggesting that the absence of *relA* mainly affects adhesion but not invasion. These results suggest that LF82 experiences amino acid limitation during infection of I-407 monolayers, but also additional stress conditions that can be partially compensated by SpoT.

As previously reported (Demarre et al., 2019), ppGpp is important for LF82’s survival inside macrophages (Fig. S2a). It is noteworthy that while we observe a reduction in survival for MG1655 ppGpp^0^ cells (2%), it seems that LF82 ppGpp° cells survive but do not replicate (100%). Considering that AIEC replicates within the phagolysosome (Lapaquette et al., 2009) we tested the ability of our strains to survive under conditions of low nutrient availability, acid conditions and oxidative stress, conditions that mimic the phagolysosome (Figs. S2b and S2c). First, we determined the ability of these strains to survive under oxidative stress induced by exposure to H_2_O_2_ (Fig. S2b). While the absence of ppGpp reduced the survival rate of MG1655, it had no effect on LF82 (Fig. S2b). Then, we grew the strains in a low nutrient medium at pH 4.5, as found inside a phagolysosome (Westman and Grinstein, 2021). We observed that while both WT strains were able to grow under these conditions, no growth was observed in the ppGpp-deficient strains (Fig. S2c). Thus, this may suggest that LF82 ppGpp° cells survive under the conditions found inside the macrophages (as WT LF82 does) but they cannot replicate due to the lack of nutrients (such as amino acids or iron).

### 3.4 The absence of ppGpp reduces the virulence of LF82 *in vivo*

Considering that ppGpp is essential for LF82 to bind to and invade epithelial cells *in vitro* (Figs. 3a and b), we decided to evaluate the changes in virulence between LF82 and LF82 ppGpp^0^ strains using the *in vivo* model of *Galleria mellonella* (Fig. 3d). This model, with cellular and humoral response, has been used to determine the virulence of different pathogen (Lucidi et al., 2019), including several strains of *E. coli* (Jønsson et al., 2017; Karczewska et al., 2023; Williamson et al., 2014). Increasing concentrations of both strains were inoculated into larvae of *G. mellonella* and the lethality recorded after 24h (Fig. 3d). As we increase the number of inoculated bacteria, we observed an increase in the number of dead larvae, but the number of bacteria required to kill them is significantly less in LF82 compared to LF82 ppGpp^0^. The LD_50_ for each strain was also calculated: 2.74·10^6^ CFU for LF82 and 7.43·10^7^ CFU for LF82 ppGpp^0^, which are significantly different (p-value < 0.01). Altogether suggest that ppGpp is involved in the virulence of LF82 in this *in vivo* model.

### 3.5 Survival when exposed to antimicrobial peptides

In the intestinal tract, bacteria are exposed to antimicrobial peptides (AMP), such as defensins, that bind to the outer membrane and create pores. Polymyxin B (PmB) and E (PmE) are commercial AMP used to treat Gram negative infections. Lysozyme is a peptide with antimicrobial properties able to degrade peptidoglycan from the cellular wall. The ability to modify the composition of the outer membrane or the presence of inhibitory factors is important for bacterial survival when exposed to these compounds. Given the role of ppGpp in lipid synthesis (My et al., 2013; Vadia et al., 2017), we determined the ability of our strains to survive exposure to different AMPs, such as polymyxin B and E, or lysozyme (Table 1). We measured the minimal inhibitory concentration (MIC) for MG1655, LF82 and their ppGpp^0^ mutants when exposed to increasing concentration of these compounds. We observed a 2-fold decrease on the MIC of MG1655 ppGpp° for PmB when compared to WT strain. No differences were observed between the LF82 WT and the ppGpp^0^ mutant, both reaching the same MIC. A similar behavior was observed for lysozyme. However, we observed a 2.5-fold decrease in the MIC for PmE in case of ppGpp-deficient strain when compared to their WT counterpart.

**Table 1:** **The effect of ppGpp on antimicrobial peptides (AMP) minimal inhibitory concentration.** The MIC of lysozyme, Polymyxin B (PmB) and Polymyxin E (PmE) of the strains MG1655, MG1655 ppGpp^0^, LF82 and LF82 ppGpp^0^. The MIC of 3 biological replicates and technical replicates is shown.

	µg/ml		
	PmB	PmE	Lysozyme
MG1655	3.13	0.21	50
MG1655 ppGpp^0^	1.56	0.09	25
LF82	3.13	0.23	50
LF82 ppGpp^0^	3.13	0.09	50

### 3.6 Gene expression changes promoted by ppGpp during infection of intestinal epithelial cells

**Fig. 4:**
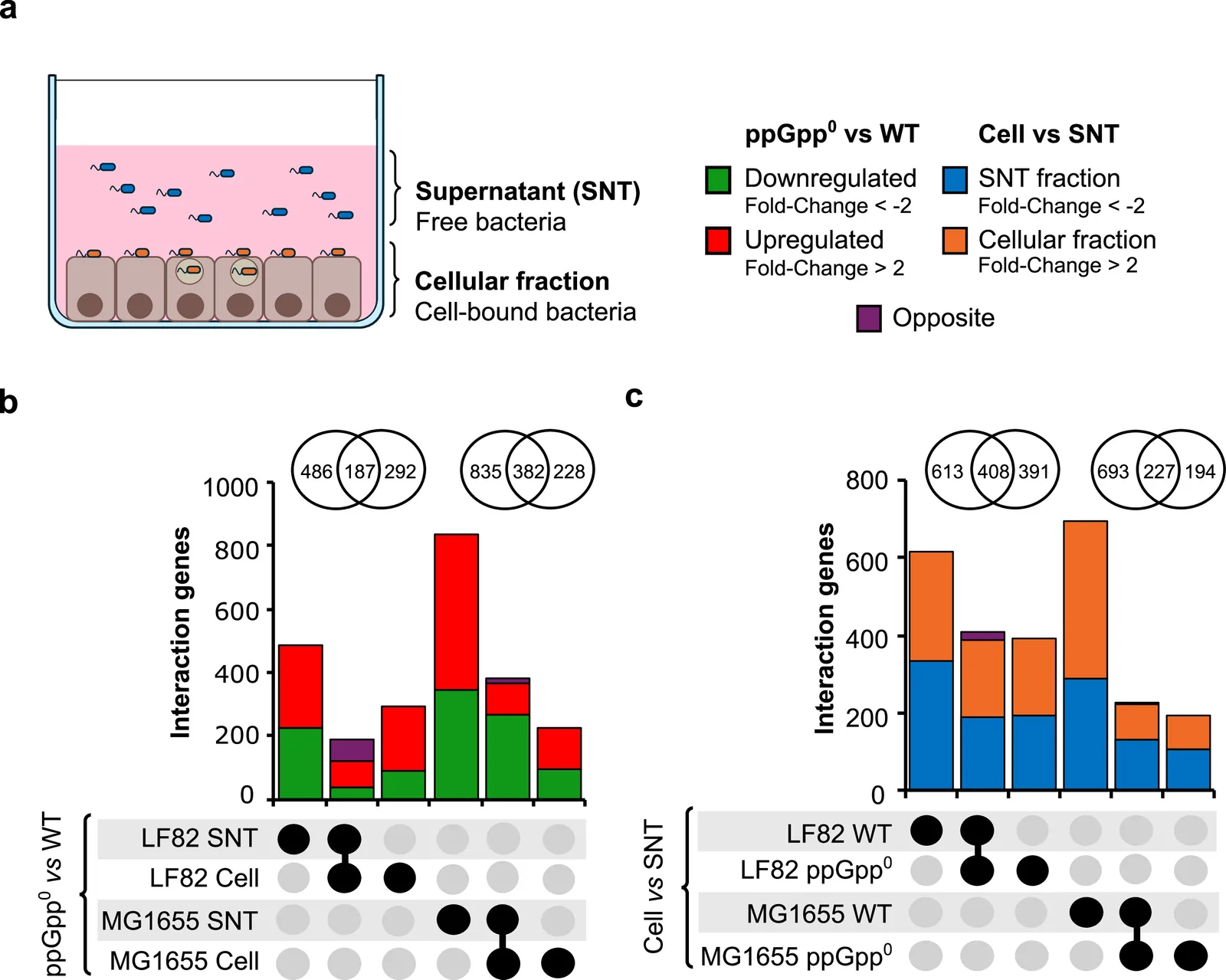
**Transcriptomic study of the effect of ppGpp during infection of epithelial cells.** a) schematic representation of our study and the comparisons performed. b) Upsets graph combined with Venn diagrams of the genes differentially expressed between the WT and the ppGpp-deficient strains comparing the common genes found in the SNT and cellular fractions. Upregulated and downregulated genes are indicated. c) Upsets graph combined with Venn diagrams of the genes differentially expressed between SNT and cellular fractions comparing the common genes found in the WT and ppGpp-deficient strain.

To investigate the role of ppGpp during AIEC’s interaction with intestinal epithelial cells, we aimed to determine gene expression patterns by RNAseq of the AIEC strain LF82, the commensal strain MG1655 and their ppGpp-deficient mutant strains in I-407 cellular cultures. Thus, RNA from infected I-407 monolayers was isolated from the supernatant of the culture flask (free bacteria, Fig. 4a) and the cellular fraction (containing bacteria adhered to and invading, Fig. 4a).

Before performing the transcriptomics study, we determined the expression of *iraP* by qPCR (Fig. S3), a gene positively regulated by ppGpp (Bougdour and Gottesman, 2007) whose expression correlates with ppGpp levels. This allowed us to follow the levels of ppGpp during the infection of epithelial intestinal cells but also confirmed that the ppGpp^0^ strains did not accumulate suppressor mutations (Murphy and Cashel, 2003) during the infection process. We observed a significant decrease in *iraP* expression in LF82 and MG1655 binding to the epithelial monolayer compared to free bacteria in the SNT (Fig. S3), suggesting a decrease on the levels of ppGpp in bacteria attached to the epithelial cells. Still, the levels of ppGpp in LF82 adhered to epithelial cells seem to be higher than in MG1655. In the absence of ppGpp, we observe a significant decrease in the levels of *iraP* in either fraction when compared to each WT strain, confirming the absence of suppressor mutations (Fig. S3).

In our transcriptomic study, two types of comparisons were made: ppGpp^0^ *vs* WT and Cellular fraction *vs* SNT (Fig. 4a). This resulted in 8 different comparisons (lists of DEGs for all 8 comparisons can be found in S4-S11 Tables). For all comparisons, we set a fold-change threshold below −2 or above 2. In the ppGpp^0^ *vs* WT comparisons, genes found upregulated (fold-change > 2) in the mutant strain suggest that these genes are repressed by ppGpp, while genes found to be downregulated (fold-change < −2) in the mutant strain suggest that these genes are activated by ppGpp. In the cellular fraction *vs* SNT comparison, genes with a fold-change above 2 are more expressed in the cellular fraction when compared to the SNT fraction, while those with a fold-change below −2 are more expressed in the SNT fraction when compared to the cellular fraction.

### 3.7 Genes regulated by ppGpp in AIEC and commensal strains

First, we compared the ppGpp-deficient mutants to each WT strain in the SNT and cellular fraction. In LF82, we observe more genes regulated by ppGpp in the SNT when compared to the cellular fraction (673 and 479 genes respectively), but only 187 are found to be regulated in both fractions (Fig. 4b). From those genes regulated by ppGpp in both fractions, 84 are found upregulated in the mutant strain (repressed by ppGpp), but in 64 genes ppGpp showed opposite roles in the different fractions: downregulated in the SNT fraction but upregulated in the cellular fraction (58 genes, Fig. S4) and *vice versa* (6 genes). Focusing on genes regulated by ppGpp only in the SNT, we observed a similar number of genes upregulated compared to the genes downregulated in the ppGpp^0^ strain (262 and 224 genes respectively). Instead, more genes were found upregulated than downregulated in the ppGpp^0^ strain in the cellular fraction (200 and 92 genes respectively). In general, our results suggest that ppGpp acts mostly as a repressor in the cellular fraction of LF82, while it has both activities in the SNT fraction (Fig. 4b). In MG1655 we observed a higher number of genes regulated by ppGpp than in LF82, but most of them are found in SNT compared to the cellular fraction (Fig. 4b, 1217 and 610 genes, respectively). From the genes regulated by ppGpp in the SNT and the cellular fraction in MG1655, 382 were found commonly regulated in both fractions, most of them downregulated in the ppGpp^0^ strain (activated by ppGpp).

**Fig. 5:**
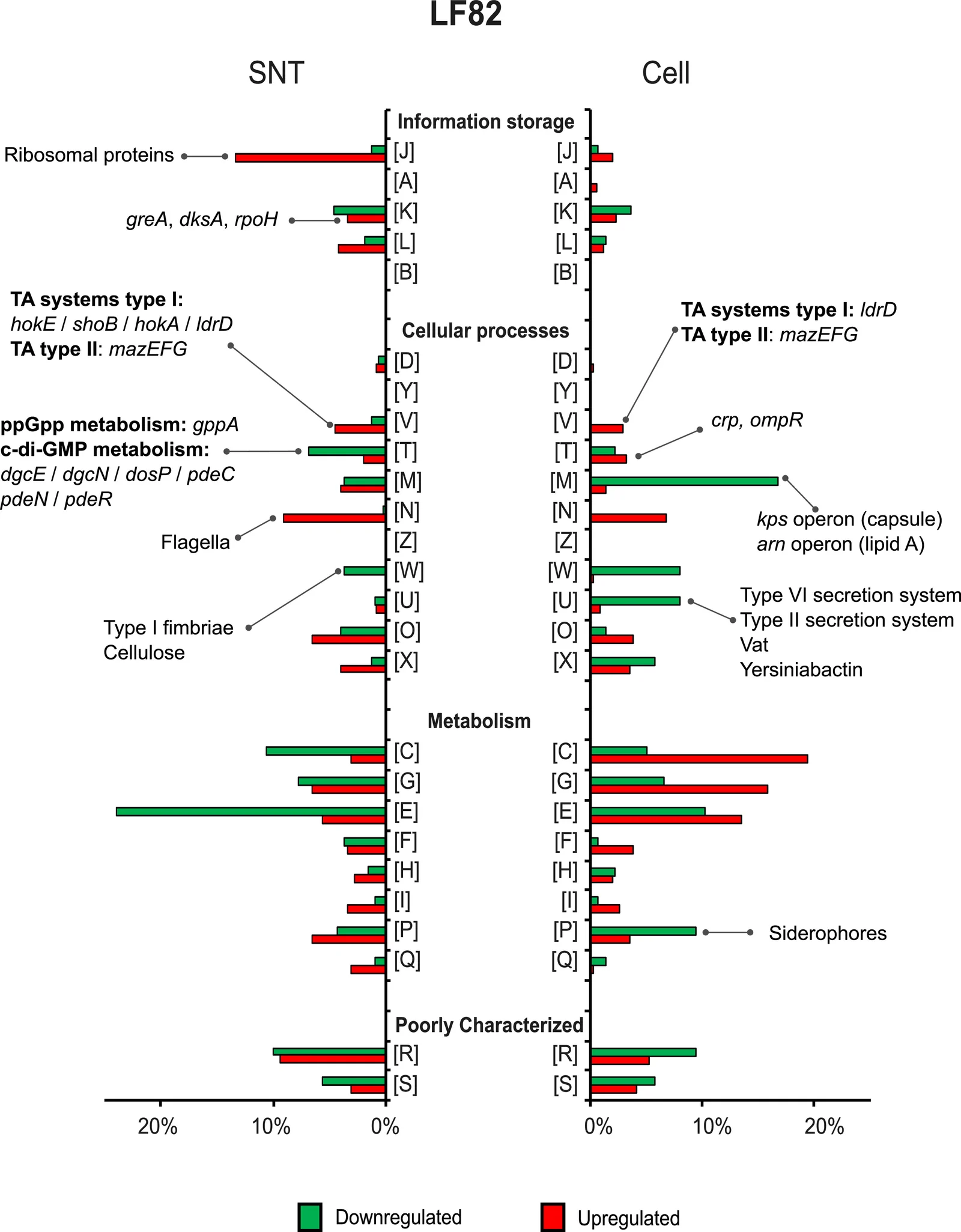
**Functional categorization of the genes differentially expressed between LF82 ppGpp^0^ and LF82 in the SNT and cellular fraction.**COG categories are: [J] Translation, ribosomal structure and biogenesis, [A] RNA processing and modification, [K] Transcription, [L] Replication, recombination and repair, [B] Chromatin structure and dynamics, [D] Cell cycle control, cell division, chromosome partitioning, [Y] Nuclear structure, [V] Defense mechanisms, [T] Signal transduction mechanisms, [M] Cell wall / membrane / envelope biogenesis, [N] Cell motility, [Z] Cytoskeleton, [W] Extracellular structures, [U] Intracellular trafficking, secretion, and vesicular transport, [O] Posttranslational modification, protein turnover, chaperones, [X] Mobilome: prophages, transposons, [C] Energy production and conversion, [G] Carbohydrate transport and metabolism, [E] Amino acid transport and metabolism, [F] Nucleotide transport and metabolism, [H] Coenzyme transport and metabolism, [I] Lipid transport and metabolism, [P] Inorganic ion transport and metabolism, [Q] Secondary metabolites biosynthesis, transport and catabolism, [R] General function prediction only, [S] Function unknown.

Regarding the functional analysis of gene expression in the SNT fraction, in MG1655 (Fig. S5) we observed that ppGpp represses gene expression of ribosomal proteins (category J), while it activates genes responsible for the biosynthesis of amino acids (category E), flagellar genes (category N), type I fimbriae and antigen 43 (category W), which is in line with previous reports (Aberg et al., 2009, 2006; Cabrer-Panes et al., 2020; Potrykus and Cashel, 2008). In LF82 (Fig. 5), we also observe that ppGpp represses genes for synthesis of ribosomal proteins (category J) and activates those for biosynthesis and transport of amino acids (category E), such as those of arginine that were found to be overexpressed in the SNT of AIEC strains compared to non-AIEC isolates (Bonet-Rossinyol et al., 2023). Type I fimbriae and the production of cellulose (category W) were also activated by ppGpp in this strain, which concur with the macrocolonies pigmentation in CR plates (Fig. 2c). No differences were observed in the expression of genes involved with production of curli, as also suggested by growth in CR plates (Fig. 2c). As expected from the motility results (Fig. 2b), the expression of flagellar genes was found to be repressed by ppGpp in LF82 (category N). The expression of catalases was downregulated in absence of ppGpp, *katG* in LF82 but *katG* and *katE* in MG1655. This difference in the expression of catalases, may explain the differential sensitivity to oxidative stress observed in Fig. S2b. Interestingly, the expression of *gppA*, that codes for the enzyme which transforms pppGpp into ppGpp, was found to be downregulated in the LF82 ppGpp^0^ strain, as well as the genes whose products are involved in the synthesis and degradation of c-di-GMP (*dcgE, dgcN, dosP, pdeC, pdeN* and *pdeR*). Several toxin-antitoxin (TA) systems (type I and II) was found to be upregulated in the LF82 ppGpp^0^ strain. The role of the TA systems in the pathogenicity of AIEC strains has been recently discussed (Bustamante et al., 2024; Bustamante and Vidal, 2020).

When assessing the genes affected by ppGpp in bacteria attached to the epithelial cells, no effect was observed on the genes coding for ribosomal proteins in either LF82 or MG1655 (Figs. 5 and S5, category J), contrary to what we previously observed in the SNT. Also, we observed in LF82 that ppGpp seems to repress the metabolic pathways genes of several amino acids (Fig. 5, category E) instead of activating them as observed in the SNT fraction. A differential effect of ppGpp on the amino acid biosynthesis genes has been previously described (Gummesson et al., 2020). We also observed that ppGpp regulates differently the expression of genes related to metabolism in LF82. While in the SNT we observed several genes whose products are involved in energy production (category C) and carbohydrate metabolism (category G) to be activated by ppGpp, they are repressed in the cellular fraction (as previously mentioned, Fig. S4). Expression of the global regulator CRP was found to be upregulated in the ppGpp-deficient strain at the cellular fraction, suggesting that its expression is repressed by ppGpp, as previously described (Johansson et al., 2000). This could explain the changes in expression of genes related to production of energy and carbohydrate metabolism (Fig. 5).

Several genes related to AIEC’s pathogenicity were downregulated in the cellular fraction of the LF82 ppGpp^0^ strain, suggesting that ppGpp activates them. The products of these genes are responsible for the synthesis of the capsule (*kps* operon), Type VI secretion system (T6SS) used to inject effector proteins into other bacteria, and the expression of Vat protein, that has been related to reorganization of the cytoskeleton of actin during infection of epithelial cells by UPEC (Díaz et al., 2020) but has been suggested to act as mucinase in AIEC (Gibold et al., 2016). We also observed downregulation of genes encoding factors involved in the synthesis of the Type II secretion system (T2SS), that have been related to transport of toxins and effectors in different pathotypes of *E. coli* (UPEC or ETEC), as well as in other pathogenic species (Korotkov et al., 2012; Kulkarni et al., 2009; Tauschek et al., 2002). Several genes encoding proteins involved in the synthesis and modification of lipopolysaccharides (LPS) are found to be downregulated in the cellular fraction of the LF82 ppGpp^0^ strain, which could explain the differences found when determining the MIC to PmE (Table 1).

### 3.8 Changes in gene expression pattern during infection

Secondly, we compared the cellular fraction to the SNT fraction for each strain (Fig. 4c). In LF82, up to, 1021 genes were dysregulated between fractions, while 799 were dysregulated in the LF82 ppGpp^0^ strain, with 408 genes found to be dysregulated in both strains. A similar number of genes was found to be overexpressed in the cellular fraction between LF82 and LF82 ppGpp^0^ strain (481 and 409 respectively), while in the SNT fraction a higher number of genes was found overexpressed in LF82 compared to LF82 ppGpp^0^ (540 and 390 respectively). In MG1655 we observe that 920 genes are dysregulated between fractions, while 421 genes are dysregulated in MG1655 ppGpp^0^, sharing 227 genes between strains. When focusing on the genes overexpressed in the cellular fraction, we observe that most are found in MG1655 compared to MG1655 ppGpp^0^ (500 and 175, respectively). We also found more genes overexpressed in the SNT at MG1655 compared to MG1655 ppGpp^0^ (420 compared to 246 genes).

**Fig. 6:**
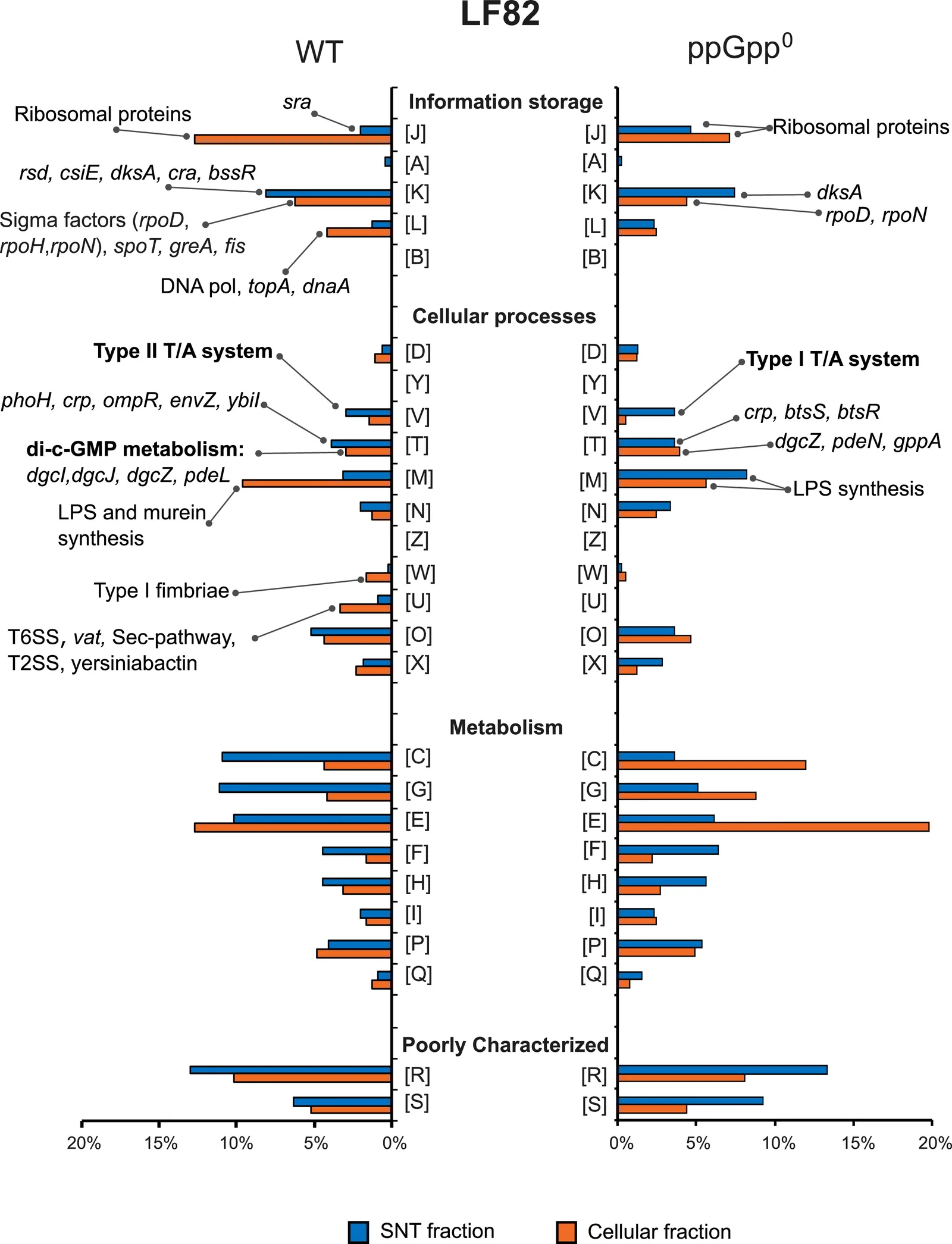
**Functional categorization of the genes differentially expressed between the cellular fraction and the SNT in strains LF82 and LF82 ppGpp^0^.**COG categories are: [J] Translation, ribosomal structure and biogenesis, [A] RNA processing and modification, [K] Transcription, [L] Replication, recombination and repair, [B] Chromatin structure and dynamics, [D] Cell cycle control, cell division, chromosome partitioning, [Y] Nuclear structure, [V] Defense mechanisms, [T] Signal transduction mechanisms, [M] Cell wall / membrane / envelope biogenesis, [N] Cell motility, [Z] Cytoskeleton, [W] Extracellular structures, [U] Intracellular trafficking, secretion, and vesicular transport, [O] Posttranslational modification, protein turnover, chaperones, [X] Mobilome: prophages, transposons, [C] Energy production and conversion, [G] Carbohydrate transport and metabolism, [E] Amino acid transport and metabolism, [F] Nucleotide transport and metabolism, [H] Coenzyme transport and metabolism, [I] Lipid transport and metabolism, [P] Inorganic ion transport and metabolism, [Q] Secondary metabolites biosynthesis, transport and catabolism, [R] General function prediction only, [S] Function unknown.

In the cellular fraction of LF82, there is an increase in the expression of the genes whose products are involved in genome replication (category L), such as initiator protein DnaA, those involved in the formation of the replisome (DNA polymerase, primase and helicase) and topoisomerase I. We also observed an increase in the expression of the genes that code for the RNA polymerase (category K) and the ribosomal proteins in the cellular fraction, compared to the SNT (Fig. 6). This suggests an increase in the bacterial replication and growth in those bacteria binding to epithelial cells, compared to those not attached. Consistent with an active replication and duplication, in the cellular fraction there is an increase in the expression of genes encoding factors involved in biosynthesis of cell wall components (category M), such as the *arn* operon and different genes whose products are involved in LPS synthesis and transport, synthesis of murein and colanic acid. In the absence of ppGpp, a dysregulation of these genes is observed, where genes important for replication, ribosomal operons and cellular envelope were found to be expressed in both fractions, consistent with ppGpp being important for the proper regulation of bacterial growth and replication (Fernández-Coll et al., 2020; Potrykus et al., 2011).

Changes in the expression of transcription regulators (category T) were also observed between the SNT and the cellular fraction. For example, *crp* and *cra* were upregulated in the SNT fraction, what may account for the differences observed in the metabolic genes (Fig. 6). As previously shown (Fig. 5), there is a change in the gene expression pattern in metabolic genes between the SNT and cellular fraction, and these changes seem to be strongly affected by ppGpp. Also, in the SNT fraction, we observed an increase in the expression of *rsd, csiE* and *sra* whose expression is mostly found during the stationary phase, suggesting that LF82 in the SNT are not growing as much as those found in the cellular fraction (as discussed above). Other global regulators, such as the complex *ompR-envZ* or *phoH* are found to be overexpressed in the SNT fraction.

Instead, in the cellular fraction there is an increase in the expression of *spoT* (synthetase and hydrolase of ppGpp) and different genes whose products are involved in the synthesis and degradation of c-di-GMP (*dgcI, dgcJ, dgcZ* and *pdeL*) suggesting that this molecule may play an important role in bacteria binding to epithelial cells. We also observed an increase in the gene expression of different sigma factors, such as σ^70^, σ^N^, σ^H^ and the σ^E^ protease (*rseP*). The expression of *rpoH* did not change between cellular fraction and SNT in the absence of ppGpp, which would reenforce the notion of ppGpp promoting a competition between sigma factors suggested by some authors (Potrykus and Cashel, 2008). It is interesting to note a change in the expression levels of several ppGpp co-factors: the expression of *dksA* is higher in the SNT fraction, while *greA* is overexpressed in the cellular fraction. These co-factors will modify the effect of ppGpp in the gene expression pattern of LF82 and could account for the differences in the role of ppGpp between both fractions.

Genes involved in secretion of proteins (category U), such as the T6SS or T2SS, and different components of the Sec system, involved in transmembrane transport of OmpA (Crane and Randall, 2017), or the Tol-Pal pathway, important for membrane stability and which seems to have a role in the formation of outer membrane vesicles (Szczepaniak et al., 2020), were upregulated in the cellular fraction. Other factors important for AIEC’s virulence, such as Vat or type I fimbriae, were also overexpressed, as expected. However, in the ppGpp^0^ strain, none of those factors were expressed in any of the fractions (Fig. 6), suggesting ppGpp is required for their expression, as previously shown (Fig. 5).

In MG1655 a similar pattern was observed (Fig. S6), showing an increased expression of genes, whose products are involved in replication and bacterial growth in the cellular fraction than in the SNT, as well as a similar change in the expression of the metabolic genes. As seen in LF82, the effects on replication and metabolism seem to be dependent on ppGpp.

### 3.9 Selection of genes that could be used as biomarkers to differentiate between AIEC and non-AIEC strains

**Fig. 7:**
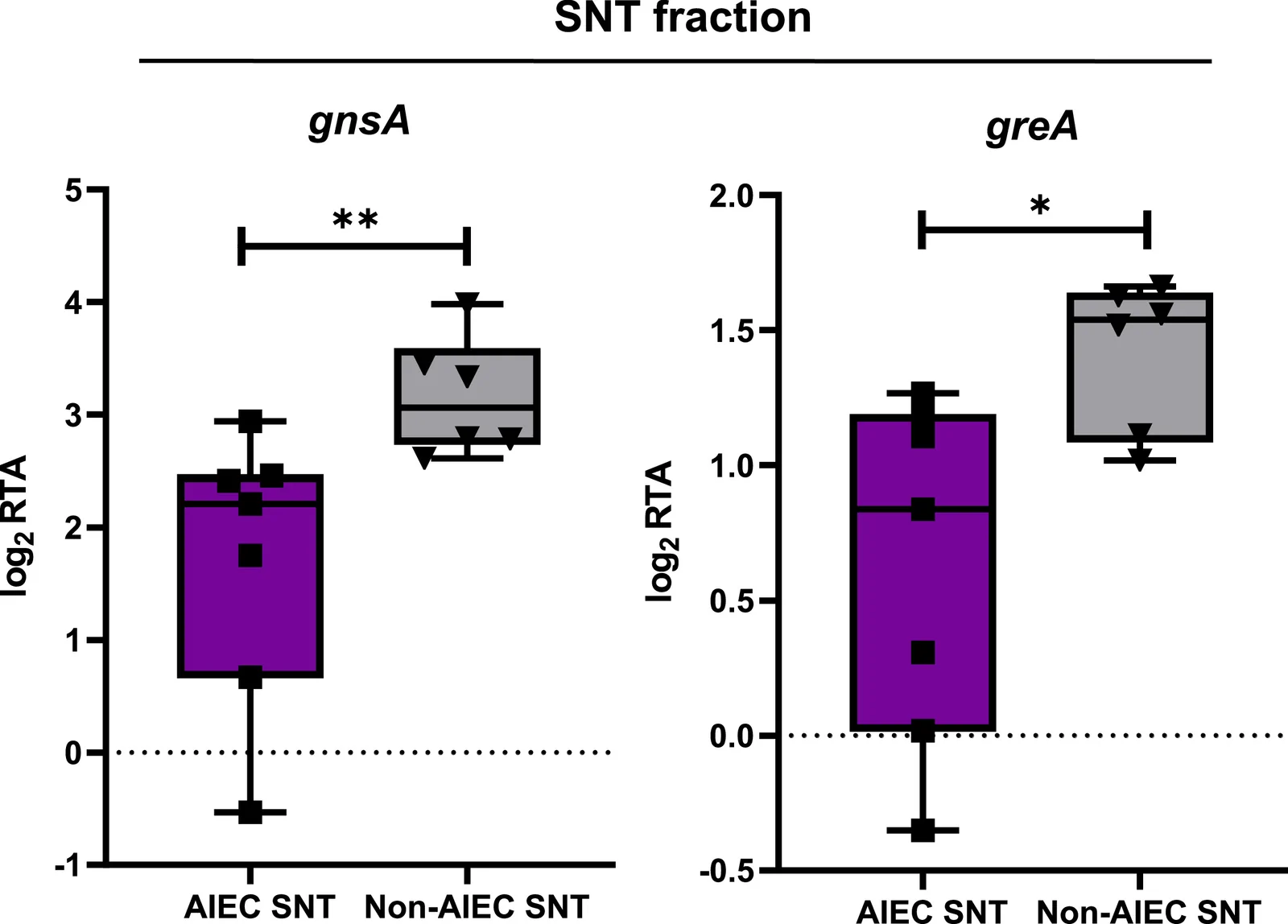
**Expression of the genes selected as putative biomarkers to separate AIEC to non-AIEC strains.** Log2 RTA values for each selected gene and condition were represented as a Boxplot with individual values for each strain. Statistical significance between groups of strains was determined with a two-tailed Mann-Whitney test (** p-value < 0.01, * p-value < 0.05).

From the RNAseq data obtained from LF82, we selected genes with a fold-change above 3 or below −3 in the comparison between WT *vs* ppGpp^0^ or between SNT *vs* Cell fraction. We decided to prioritize those genes that showed differential behavior in MG1655, selecting up to 64 genes. We also included 18 genes relevant for our research group, related to AIEC’s pathogenicity (Barnich et al., 2025) or ppGpp’s synthesis and activity, creating a list of 82 genes of possible biomarkers. The expression of these genes was determined using RNA isolated from the SNT and cellular fraction of a collection of 13 strains (7 AIEC and 6 non-AIEC), used in a previous study (Bonet-Rossinyol et al., 2023). Significant differences between AIEC and non-AIEC groups were assessed for each fraction, as well as between fractions (Table S12), using a Kruskal-Wallis analysis. No differences between AIEC and non-AIEC were observed in either fraction. In contrast, several genes showed significant differences between fractions in both groups of strains. We then selected those genes that showed significant differences between fractions in one group of strains but not in the other, suggesting group-specific expression patterns. For those genes, statistical significance between AIEC and non-AIEC strains was evaluated using a two-tailed Mann-Whitney test. Two genes (*gnsA* and *greA*) were selected. The expression of both genes was found increased in the non-AIEC group of strains compared to the AIEC in the SNT fraction (Fig. 7). To test their potential as molecular biomarkers, a binary logistic regression was performed (Table 2).

**Table 2:** **Sensitivity and specificity of AIEC putative molecular biomarkers.** Binary logistic regression was performed to calculate sensitivity, specificity, and accuracy for each biomarker, individually and in combination, using 7 AIEC and 6 non-AIEC strains. Model fit was assessed using the Nagelkerke R^2^.

Biomarker	Threshold	Sensitivity	Specificity	Accuracy	Nagelkerke R^2^
*gnsA*	0.67	85.71%	100.00%	92.31%	0.712
*greA*	0.25	100.00%	66.67%	84.61%	0.625
Combination					
*greA* + *gnsA*	0.50	100.00%	100.00%	100.00%	1.000

From the putative biomarkers, *gnsA* has accuracy, sensitivity and specificity above 80%. Combination of both genes (*gnsA* and *greA*) increases them to a 100%, with a R^2^ of the model up to 1.

## 4 Discussion

AIEC strains share no unique common virulence factors associated, and they are genetically similar to commensal strains, bringing us to conclude a differential regulation in AIEC compared to non-AIEC. Considering the role of the global regulator ppGpp in the pathogenicity in several bacteria (Fernández-Coll and Cashel, 2020), we hypothesized that ppGpp-dependent genes would give us a better understanding of the pathogenicity of AIEC strains, as well as highlighting genes that could be targeted as biomarkers.The study presented here shows that in the absence of ppGpp, LF82 loses its pathogenic traits, rendering it unable to adhere to or to invade intestinal epithelial cells, as well as reduces its virulence in the *in vivo* model of *G. mellonella* (Fig. 3). All these phenotypical effects are accompanied by a myriad of changes in gene expression during infection of epithelial cells that could explain the observed phenotypes (Fig. 5, Fig. 6).

Upon the infectious process, we observed an important change in bacterial transcriptional profile between the cellular fraction and the SNT. Bacteria attached to epithelial cells showed lower levels of ppGpp (Fig. S3), together with increased expression of ribosomal operons and replication machinery, suggesting that the host cell surface provides a more favorable environment for bacterial growth. Consistently, the requirement of RelA for proper adhesion to the cellular monolayer, but not for invasion (Fig. S1), supports the idea that bacteria in the SNT experience amino acid starvation. In line with this, LF82 showed increased expression of *rsd* (anti-σ^70^ factor) and *sra* (a ribosomal-associated protein during stationary phase) in the SNT, suggesting a transition towards stationary phase under these conditions. It should be noted that the epithelial monolayer does not represent a passive surface for bacterial attachment but rather a dynamic interface capable of modulating the local environment, including the release of compounds that may serve as nutrients (Schwärzler et al., 2024). This could generate a local nutrient gradient between the SNT fraction and the cellular surface, potentially explaining the differences observed between both fractions. Importantly, the fact that ppGpp did not have any effect on the expression of ribosomal proteins in the cells attached to the cellular monolayer (Fig. 5) is consistent with the well-established role of ppGpp as a central regulator of ribosomal synthesis and DNA replication during nutrient limitations (Fernández-Coll et al., 2020; Potrykus et al., 2011).

In MG1655, the expression of Type I fimbriae genes correlates with the ability to form biofilm under the conditions tested (Fig. 2b), but not in LF82. It is important to note that LF82 possesses other adhesins than Type I fimbriae (*auf, lfpA, yqi* or curli), that were not found to be affected by ppGpp in our transcriptomic analysis, suggesting that the increase in biofilm formation in the absence of ppGpp under the conditions tested, may be an indirect effect. Adhesion of LF82 to intestinal epithelial cells has been linked to the presence of Type I fimbriae (Boudeau et al., 2001), and the downregulation of their expression observed in the ppGpp^0^ strain (Fig. 5) would explain the decrease in adhesion observed in Fig. 3a. Consistently, ppGpp-deficient strains show negative aggregation in the MSYA assay (Fig. 2a), indicating the absence of functional Type I fimbriae. There is also a decrease in expression of genes involved in cellulose production in the absence of ppGpp, which is important for bacterial adhesion to epithelial cells together with other extracellular matrix components (Hollenbeck et al., 2018), but also for the survival inside of phagolysosome (Prudent et al., 2021). Another factor related to LF82’s survival inside the phagolysosome is the siderophore yersiniabactin (encoded by the genes *irp1* and *irp2*) and has been observed to be regulated by ppGpp (Fig. 5), as well as other siderophores as *fhuF* or *fecD*.

Moreover, we must highlight that gene expression of several hydrolases and synthetases of the secondary messenger c-di-GMP was found to be downregulated by ppGpp in the SNT fraction (Fig. 5, Tables S4-S7) while others are found to be upregulated in the cellular fraction in LF82 and LF82 ppGpp^0^ (Fig. 6, Tables S8-S11). These hydrolases and synthetases bind to different factors involved in the production of biofilm and modify the levels of c-di-GMP at a local level (Hengge, 2021). One of these factors is BcsB, responsible for the control of cellulose synthesis depending on c-di-GMP (Richter et al., 2020), whose expression has been found to be downregulated in the absence of ppGpp in LF82 (Fig. 5, Tables S4–5).

Several known mechanisms of pathogenicity of LF82 have been shown to be regulated by ppGpp in cells attached to the cellular fraction (Fig. 5) during this study. However, up to 17% of the genes affected by ppGpp in LF82 have unknown functions (category S) or they are poorly characterized (category R), which highlights the need for a better understanding of AIEC pathogenicity mechanisms and strategies.

We observed changes in the expression of ppGpp co-factors: *dksA* expression was found to be increased in the SNT fraction of LF82, while *greA* was found to be increased in the cellular fraction. Both proteins bind to the secondary channel of the RNA polymerase (Laptenko et al., 2003; Perederina et al., 2004). A competition between them was suggested (Fernández-Coll et al., 2018; Vinella et al., 2012; Zenkin and Yuzenkova, 2015), and the changes in gene expression observed here reinforce this idea. In addition, the activity and conformation of these factors may be modulated by environmental factors such as oxidative stress and low pH (Furman et al., 2015; Henard et al., 2014), which are likely encountered by LF82 during host interaction (Lapaquette et al., 2009). ppGpp binds to the secondary channel of the RNA polymerase together with DksA (Molodtsov et al., 2018; Ross et al., 2016), and this interaction may differ when GreA binds in the same site. Thus, the differential expression of *dksA* and *greA* between fractions could favor an exchange in the occupancy of the secondary channel of the RNA polymerase, contributing to the distinct roles of ppGpp between fractions. It is important to note that in the collection of strains used here, the expression of *greA* in the SNT was found to be higher in the non-AIEC strains than in AIEC strains (Fig. 7), which could be used as possible AIEC biomarker together with the expression of *gnsA* (Table 2).

The expression levels of different genes and combination of them (Table 2), showed a promising possibility to differentiate between AIEC and non-AIEC strains using a collection of 7 AIEC strains and 6 non-AIEC strains. Further studies, using more extensive collections of strains from different geographical locations are required to make a definitive conclusion. At the same time, studies of expression of these genes under more physiological conditions, such as tissue biopsies or stool samples, would be paramount to bring these biomarkers to clinical settings.

## Funding

This study was supported by the Spanish Ministry of Science, Innovation and Universities under Grants PID2021–126699NB-100 and PID2024–159916NB-I00; and the Catalan Agency for Management of University and Research Grants (AGAUR) under Grant, 2021 BP 00087.

## CRediT authorship contribution statement

**Llorenç Fernández-Coll:** Conceptualization, Methodology, Formal analysis, Investigation, Validation, Writing – original draft, Writing – review & editing, Visualization, Supervision, Funding acquisition. **Queralt Bonet-Rossinyol:** Investigation, Formal analysis, Writing – review & editing. **Mireia López-Siles:** Formal analysis, Investigation, Validation, Funding acquisition, Resources, Writing – review & editing. **Katarzyna Potrykus:** Formal analysis, Investigation, Validation, Resources, Writing – review & editing. **Maria Núria Ramos-Corominas:** Investigation, Formal analysis, Writing – review & editing. **Margarita Martinez-Medina:** Conceptualization, Methodology, Resources, Writing – review & editing, Supervision, Funding acquisition.

## Declaration of competing interest

The authors declare the following financial interests/personal relationships which may be considered as potential competing interests:Llorenc Fernandez-Coll, Maria Nuria Ramos-Corominas, Mireia Lopez-Siles and Margarita Martinez-Medina patent BIOMARKERS FOR THE DETECTION OF ADHERENT-INVASIVE *E. coli* (AIEC) STRAINS issued to EP26382726.3. If there are other authors, they declare that they have no known competing financial interests or personal relationships that could have appeared to influence the work reported in this paper.

## Appendix Supplementary materials

Image, application 1

Image, application 2
