# Supplementary material for: Changes in ppGpp levels impact gene expression and virulence features of Adherent-Invasive Escherichia coli strain LF82
Source: Curr Res Microb Sci. 2026 Jul 7;11:100641. doi: 10.1016/j.crmicr.2026.100641 (PMC13356692; doi:10.1016/j.crmicr.2026.100641)
Supplement: Supplementary file 1 [file mmc1.docx]

| **Strains:** | | | |
| --- | --- | --- | --- |
| **Name** | **Description** | **Reference** |  |
| LF82 | O83:H1, *ampC, ibeA, fimH, fimA_vMT78_*_._ Phylogroup B2. Isolated from a chronic ileal lesion of a patient with CD | (Darfeuille-Michaud et al., 1998) |  |
| MG1655 | OR:H48, F-, *ilvG*, *rph1.* Phylogroup A | (Clermont et al., 2000) |  |
| LF82 ppGpp^0^ | LF82 Δ*relA*::Km^R^ Δ*spoT*::Cm^R^ | This study |  |
| MG1655 ppGpp^0^ | MG1655 Δ*relA*::Km^R^ Δ*spoT*::Cm^R^ | This study |  |
| **Plasmids:** | | |  |
| **Name** | **Description** | **Reference** |  |
| pKD46 | *aac*(3)*-Id* *araC*-P_araB_-γβ exo oriR101 repA101ts | (Doublet et al., 2008) |  |
| pKD3 | *bla* FRT *cat* FRT PS1 PS2 oriRγ | (Datsenko and Wanner, 2000) |  |
| pKD4 | *bla* FRT *aph* FRT PS1 PS2 oriRγ | (Datsenko and Wanner, 2000) |  |

**Table S1. List of strains and plasmids used in this study.**

**Table S3. Bacterial growth rate.** Specific growth rate (µ) quantifies the population growth per unit time, being inversely related to the generation time (units: hours^-1^). Mean and standard deviation of 3 biological replicates is shown. Cultures grown in LB and M9 supplemented with 0.2 % glucose were grown at 37ºC with constant agitation at 200 rpm, while MEM cultures were grown in static and in 5% CO_2_ incubator.

|  |  | LB | M9 with glucose | MEM |
| --- | --- | --- | --- | --- |
| MG1655 | WT | 0.96 h^-1^ ± 0.06 | 0.52 h^-1^ ± 0.03 | 0.18 h^-1^ ± 0.02 |
|  | ppGpp^0^ | 0.96 h^-1^ ± 0.09 | No growth | 0.20 h^-1^ ± 0.02 |
| LF82 | WT | 1.29 h^-1^ ± 0.1 | 0.52 h^-1^ ± 0.14 | 0.32 h^-1^ ± 0.05 |
|  | ppGpp^0^ | 1.23 h^-1^ ± 0.08 | No growth | 0.26 h^-1^ ± 0.04 |


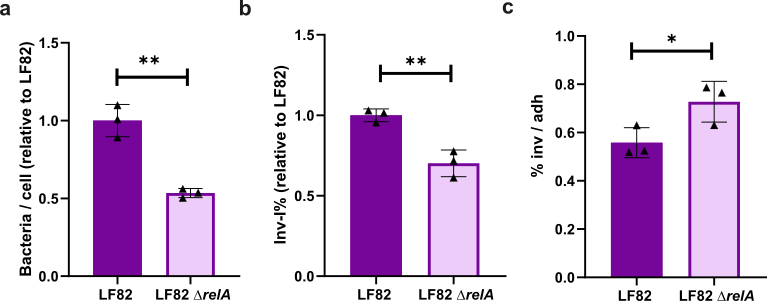


**Fig. S1. Effect of *relA* deletion in the ability to adhere to and invade epithelial cells I-407.** Ability of the strains LF82 and LF82 Δ*relA* to adhere to a) and invade b) epithelial intestinal cells was determined. c) Percentage of bacteria invading I-407 relative to those able to adhere to epithelial cells. Mean (with data points) and standard error (SEM) of 3 biological replicates are represented. A T-test was used to determine those values significantly differently (** p-value < 0.01, * p-value < 0.05).


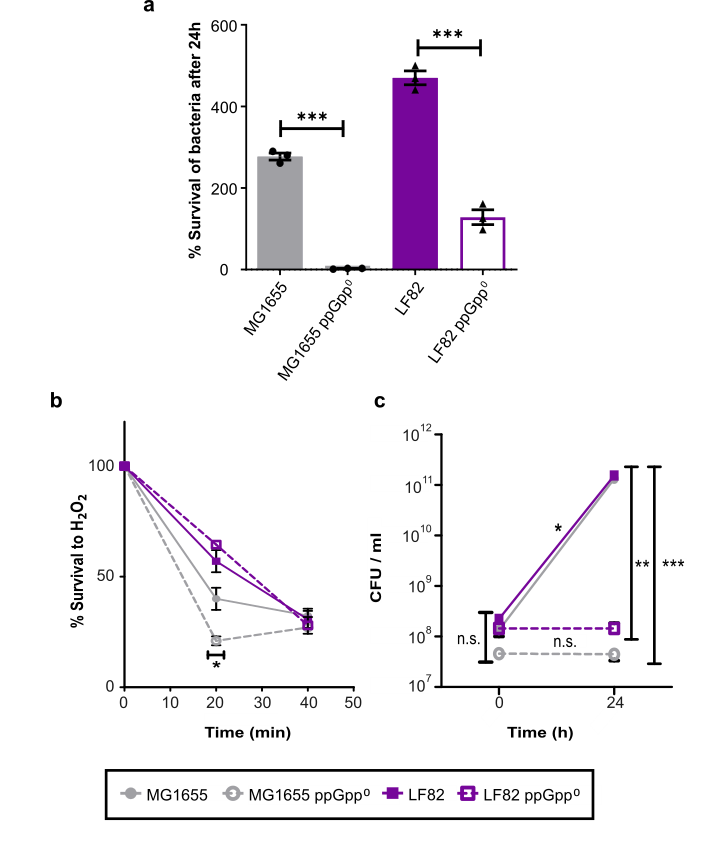


**Fig. S2. Survival of LF82, MG1655 and their ppGpp^0^ mutants to macrophages and the environmental conditions found inside the phagolysosome.** a) Ability of the strains MG1655, MG1655 ppGpp^0^, LF82 and LF82 ppGpp^0^ to survive within J774 macrophages. b) Survival to oxidative stress using 2mM of H_2_O_2_. Data is represented as percentage relative to the number of bacteria before adding H_2_O_2_. c) Strains MG1655, MG1655 ppGpp^0^, LF82 and LF82 ppGpp^0^ were grown in acid and nutrient-poor medium and CFU/ml was determined at time 1 and 24 hours. A T-test was used to determine those values significantly different, but Welch correction was applied to panel a. The mean and standard error (SEM) from 3 biological replicates is represented. (*** p-value < 0.001, ** p-value < 0.01, * p-value < 0.05, n.s. p-value > 0.05).





**Fig. S3. Extrapolation of the levels of ppGpp during the invasion of the epithelial cell model through the expression of *iraP*.** The expression of *iraP*, that correlates with the levels of ppGpp, was determined by RT-qPCR in the strains MG1655, MG1655 ppGpp^0^, LF82 and LF82 ppGpp^0^ in the SNT fraction and the cellular fraction after infection of intestinal epithelial cells (I-407). Mean and standard error (SEM) of 3 biological replicates and 3 technical replicates is represented. A T-test was used to determine those values significantly different (** p-value < 0.01, * p-value < 0.05, n.s. p-value > 0.05).


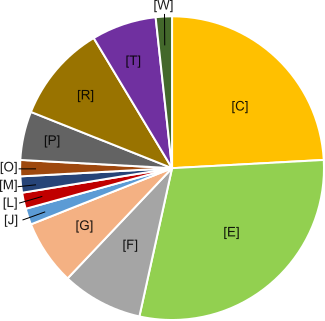


**Fig. S4. Functional categorization of those genes found down-regulated in the LF82 ppGpp^0^ strain at the SNT fraction, but up-regulated in the cellular fraction.** COG categories are: [J] Translation, ribosomal structure and biogenesis, [L] Replication, recombination and repair, [T] Signal transduction mechanisms, [M] Cell wall / membrane / envelope biogenesis, [W] Extracellular structures, [O] Posttranslational modification, protein turnover, chaperones, [C] Energy production and conversion, [G] Carbohydrate transport and metabolism, [E] Amino acid transport and metabolism,[F] Nucleotide transport and metabolism, [P] Inorganic ion transport and metabolism [R] General function prediction only.


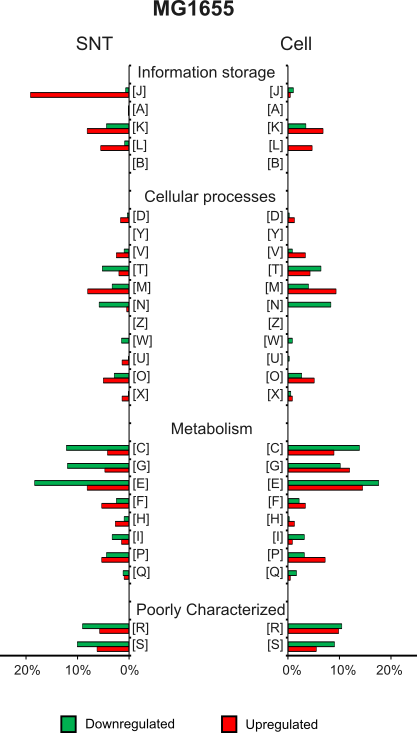


**Fig. S5. Functional categorization of the genes differentially expressed between MG1655 ppGpp^0^ and MG1655 in the SNT and cellular fraction.**COG categories are: [J] Translation, ribosomal structure and biogenesis, [A] RNA processing and modification, [K] Transcription, [L] Replication, recombination and repair, [B] Chromatin structure and dynamics, [D] Cell cycle control, cell division, chromosome partitioning, [Y] Nuclear structure, [V] Defense mechanisms, [T] Signal transduction mechanisms, [M] Cell wall / membrane / envelope biogenesis, [N] Cell motility, [Z] Cytoskeleton, [W] Extracellular structures, [U] Intracellular trafficking, secretion, and vesicular transport, [O] Posttranslational modification, protein turnover, chaperones, [X] Mobilome: prophages, transposons, [C] Energy production and conversion, [G] Carbohydrate transport and metabolism, [E] Amino acid transport and metabolism,[F] Nucleotide transport and metabolism, [H] Coenzyme transport and metabolism, [I] Lipid transport and metabolism, [P] Inorganic ion transport and metabolism, [Q] Secondary metabolites biosynthesis, transport and catabolism, [R] General function prediction only, [S] Function unknown.


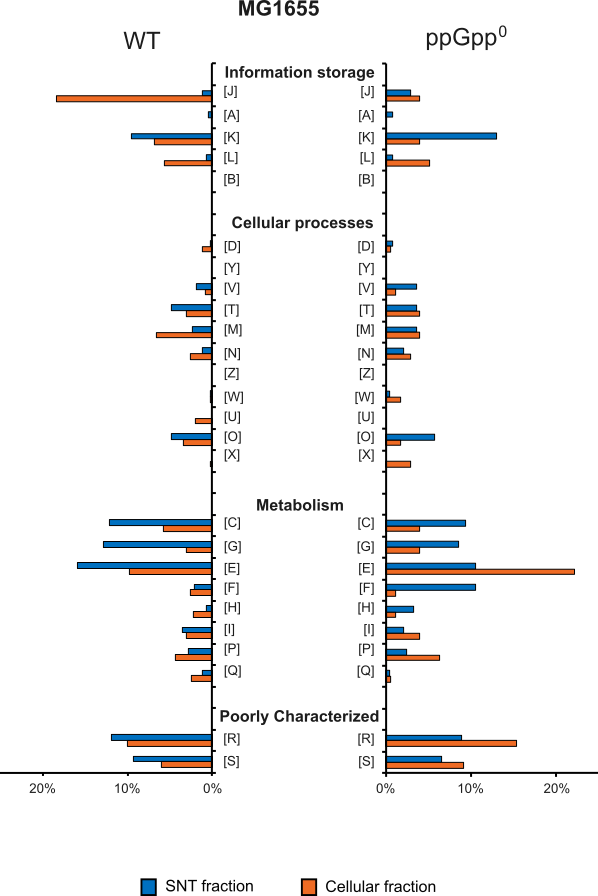


**Fig. S6. Functional categorization of the genes differentially expressed between the cellular fraction and the SNT in strains MG1655 and MG1655 ppGpp^0^.** COG categories are: [J] Translation, ribosomal structure and biogenesis, [A] RNA processing and modification, [K] Transcription, [L] Replication, recombination and repair, [B] Chromatin structure and dynamics, [D] Cell cycle control, cell division, chromosome partitioning, [Y] Nuclear structure, [V] Defense mechanisms, [T] Signal transduction mechanisms, [M] Cell wall / membrane / envelope biogenesis, [N] Cell motility, [Z] Cytoskeleton, [W] Extracellular structures, [U] Intracellular trafficking, secretion, and vesicular transport, [O] Posttranslational modification, protein turnover, chaperones, [X] Mobilome: prophages, transposons, [C] Energy production and conversion, [G] Carbohydrate transport and metabolism, [E] Amino acid transport and metabolism, [F] Nucleotide transport and metabolism, [H] Coenzyme transport and metabolism, [I] Lipid transport and metabolism, [P] Inorganic ion transport and metabolism, [Q] Secondary metabolites biosynthesis, transport and catabolism, [R] General function prediction only, [S] Function unknown.


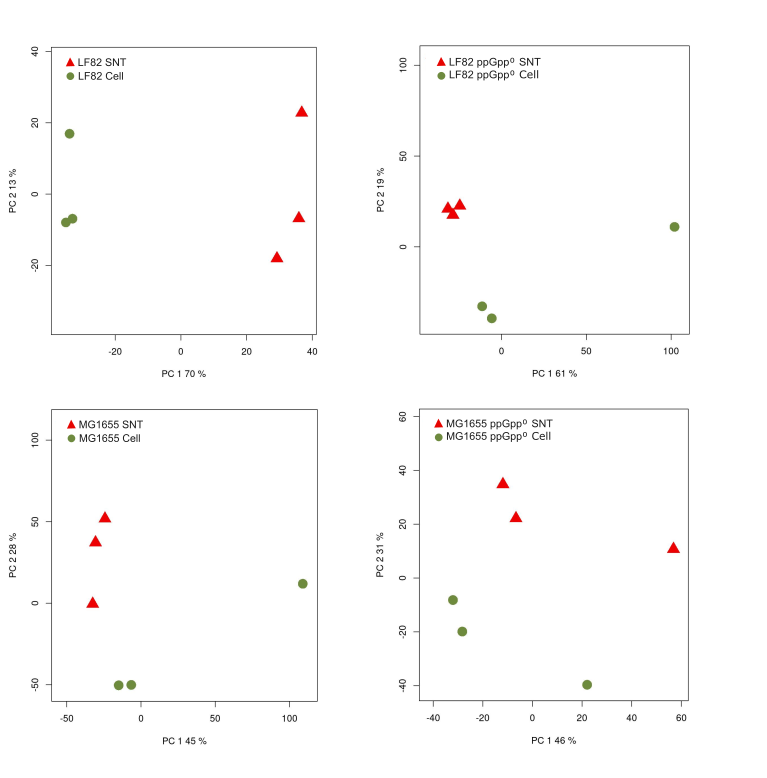
**Fig. S7. Principal component analysis (PCA) of the different samples analyzed by RNAseq comparing the SNT and the cellular fraction.**

**References**

Clermont, O., Bonacorsi, S., Bingen, E., 2000. Rapid and simple determination of the Escherichia coli phylogenetic group. Appl. Environ. Microbiol. 66. https://doi.org/10.1128/AEM.66.10.4555-4558.2000

Darfeuille-Michaud, A., Neut, C., Barnich, N., Lederman, E., Di Martino, P., Desreumaux, P., Gambiez, L., Joly, B., Cortot, A., Colombel, J.F., 1998. Presence of adherent Escherichia coli strains in ileal mucosa of patients with Crohn’s disease. Gastroenterology 115. https://doi.org/10.1016/S0016-5085(98)70019-8

Datsenko, K.A., Wanner, B.L., 2000. One-step inactivation of chromosomal genes in Escherichia coli K-12 using PCR products. Proc. Natl. Acad. Sci. U. S. A. 97, 6640–5. https://doi.org/10.1073/pnas.120163297

Doublet, B., Douard, G., Targant, H., Meunier, D., Madec, J.Y., Cloeckaert, A., 2008. Antibiotic marker modifications of λ Red and FLP helper plasmids, pKD46 and pCP20, for inactivation of chromosomal genes using PCR products in multidrug-resistant strains. J. Microbiol. Methods 75. https://doi.org/10.1016/j.mimet.2008.06.010
